# Nonlinear active disturbance rejection mechanism based sliding mode control for enhancing electric power assisted steering performance

**DOI:** 10.1371/journal.pone.0321664

**Published:** 2025-04-11

**Authors:** Tuan Anh Nguyen, Tung Lam Nguyen

**Affiliations:** 1 Faculty of Mechanical Engineering, Thuyloi University, Hanoi, Vietnam; 2 School of Electrical and Electronic Engineering, Hanoi University of Science and Technology, Hanoi, Vietnam; National University of Singapore, SINGAPORE

## Abstract

Electric Power Assisted Steering (EPAS) systems provide vehicle stability and safety under various driving conditions. Previous studies often applied only one or several traditional algorithms to control the performance of EPAS systems and ignored the influence of external disturbances. This increases the signal tracking error and causes other adverse effects on the system. In this article, we propose designing a nonlinear robust control mechanism that combines Sliding Mode Control (SMC) and Nonlinear Active Disturbance Rejection Control (NADRC) techniques to solve the existing issues. The article’s novelty lies in utilizing a Nonlinear Extended State Observer (NESO) and Nonlinear Tracking Differentiator (NTD) to improve the performance of the proposed control mechanism. In addition, ideal assisted characteristic curves have been innovated based on nonlinear functions to improve the vehicle’s driving comfort and stability, which is considered the second new contribution. The simulation results show that most of the steady-state errors of the proposed controller are only about 2% (*v*_1_ =  30 km/h) and no more than 3.5% (*v*_2_ =  70 km/h) except for steering motor current. The observed errors of the state variables are less than 1.4%, while the disturbance error is only about 6.9%. Finally, it is claimed that common issues like overshoot, chattering, and sensor noise do not affect the EPAS system when the proposed method is used to control it.

## 1. Introduction

Power-Assisted Steering (PAS) was invented about 7 decades ago to assist in steering. Over the years of development, PAS systems have undergone continuous improvement and upgrades, striving for operational convenience and safety. Today, PAS systems are equipped on all cars. While heavy trucks often use Hydraulic Power Assisted Steering (HPAS) or Electrohydraulic Power Assisted Steering (EHPAS) with a recirculating ball mechanism, most passenger cars are equipped with Electric Power Assisted Steering (EPAS) with a rack and pinion mechanism [1]. According to Hartono et al., EPAS is operated through a Permanent Magnet Synchronous Motor (PMSM) or Brushless Direct Current Motor (BLDCM), which is considered an actuator of the system [2]. In [3], Nguyen and Nguyen argued that the EPAS system offers many advantages over the HPAS or EHPAS systems, including a simple structure, lightweight, and environmental friendliness. A study conducted by Park et al. confirmed that EPAS systems have lower fuel consumption than conventional hydraulic steering systems [4]. HPAS and EHPAS systems operate continuously even when not steering, causing vibration and noise due to the hydraulic pump drive and affecting comfort [5]. In contrast, the EPAS system is only activated under certain conditions and operates smoothly.

### 1.1. EPAS control overview

Although EPAS systems have been used since the beginning of the 21^st^ century, the number of studies on system control is still limited. Many different things affect how control algorithms are designed, such as control targets, control objects, mathematical models of the system, the effect of disturbances and uncertainties, and more.

Some standard control methods for linear systems, like Proportional-Integral-Derivative (PID) and Linear Quadratic Regulator (LQR), have been used in several new studies. In [6], Hassan et al. presented the design of a simple PID controller for controlling the EPAS system, where the actuator is mounted at the steering column. They used the Genetic Algorithm (GA) to tune the control parameters instead of conventional methods, such as trial and error or pole placement. The structure of the GA presented in [6] is quite simple, with an objective function formulated based on minimizing the Mean Square Error (MSE). Hanifah et al. designed Particle Swarm Optimization (PSO) and Ant Colony Optimization (ACO) methods to find the optimal PID control parameters [7]. The calculations in [7] show that using the PSO and ACO methods instead of regular PID cuts the electric motor’s power use by 0.82% and 7.41%, respectively. In [8], Zheng and Wei designed a fuzzy technique to dynamically tune the PID controller’s parameters. The simulations show that when fuzzy PID is used instead of classical PID, the yaw rate response overshoot decreases from 14.4% to 3.9%. Another study by Zhao et al. backs up this idea, showing that the H_ ∞_ control has a better step response in both the frequency and time domains than the PID control [9]. The overshoot occurs when the PID controller controls the EPAS system under abrupt excitations [10]. The steady-state error of the PD controller is quite large, causing performance degradation in the control of the automotive steering system [11]. In general, the PID control technique is quite simple and is often used to generate reference signals for the system [12]. This control method is only suitable for simple linear systems with Single Input and Single Output (SISO) and is unaffected by disturbance and uncertainty. Regarding systems with Multiple Inputs and Multiple Outputs (MIMO), choosing the LQR technique is a highly efficient solution, unlike the conventional PID [13]. Chitu et al. [14] developed an LQR controller for the EPAS system that uses a single pinion and rack mechanism to lower the cost function. However, the results reported in [14] are not close to reality when they examined the operation of a car at extremely high speed (*v* =  240 km/h). Liu et al. utilized GA to optimize the parameters for LQR control and improve the control performance [15]. However, the LQR technique is only applicable to simple linear systems. This method fails to control complex systems. It also makes the steady-state error bigger because of sensor noise, which happens when the sensor directly picks up multiple signals [16].

Several robust control techniques have been utilized to control the performance of EPAS systems to address the issues related to nonlinearity, uncertainty, and disturbance. In [17], Nguyen and Iqbal designed an integrated scheme based on Backstepping Control (BSC). The input signal of the BSC is adjusted through the PID controller, whose parameters are optimized by the GA over 1000 generations. The results in [17] show that the single BSC has a significant steady-state error, but the integrated controller (BSPID-GA) has much less error. Instead of finding fixed control parameters using the GA, Nguyen proposed utilizing the fuzzy algorithm to dynamically adjust these values to improve the system’s adaptability [18]. A combination of fuzzy and BSC techniques for controlling automotive steering systems was presented in [19]. However, the motor current’s Root Mean Square (RMS) error is significant. The integrated controllers introduced in [17–19] only apply to control linear dynamic models. Nguyen developed adaptive controllers for complex nonlinear dynamic models in [20,21]. These controllers used a mix of PID, BSC, and fuzzy techniques. Although the Lyapunov criterion guarantees the stability of the above controllers, some simplifications in the calculation lead to practical errors.

Disturbance has many adverse effects on the system, leading to increased steady-state error. In [22], Ma et al. designed a control mechanism called Active Disturbance Rejection Control (ADRC) to eliminate the influence of unwanted disturbances and improve the EPAS system’s performance. The structure of the controller introduced in [22] includes a Tracking Differentiator (TD), an Extended State Observer (ESO), and a state feedback rule. However, the lack of a comparison of results hinders evaluating the ADRC scheme’s performance. According to Zheng and Wei, ADRC is highly efficient under severe working conditions. The simulation results presented in [23] confirmed this. However, the influence of chattering and overshoot phenomena still exists. Formulating TD and ESO according to linear mathematical models will increase the system’s steady-state error [24].

For uncertain nonlinear systems that are affected by disturbances, Sliding Mode Control (SMC) is a control solution that provides high performance. In [25], Marouf et al. designed a second-order SMC mechanism by selecting a sliding surface and control law to apply to the EPAS system. Kim et al. experimented to validate the performance of SMC for the automotive steering system [26]. Other influences are considered disturbances and are estimated by a Disturbance Observer (DO). However, the algorithm model proposed in [26] is not specifically provided. Chattering is a common problem that the SMC mechanism has to deal with and is hard to eliminate, which lowers the system’s performance [27,28]. In [29], Lu et al. designed an adaptive fuzzy SMC to solve the chattering phenomenon. They used the sliding surface and its derivative as inputs to the fuzzy controller. Kim et al. made an ESO that works with the SMC mechanism to figure out the change of state variables [30]. This was done to get rid of the effect of sensor noise. Instead of using the ESO, Li et al. utilized a Kalman filter to observe the disturbance [31]. Although observers and filters partially reduce the effect of chattering, it cannot be completely eliminated. In [32,33], Nguyen and Iqbal designed an integrated control mechanism that combines PID and SMC, where fuzzy and GA methods tune the control parameters. However, the system characteristics are not fully described. In addition, the effect of disturbance is assumed to be known for sinusoidal, square wave, or random signals. This is not suitable for practical applications. A combination of SMC and BSC for improving control performance was proposed in [34]. The control signal selection only ensures the stability of the component controllers, which is considered a significant limitation. Overall, the effect of chattering still exists and causes some negative impacts on the system.

Regarding MIMO systems, the system output signals (changes in state variables) are utilized as input signals for controllers. Directly measuring state variables with physical sensors causes sensor noise and is costly. In addition, measuring the effects of external disturbances is difficult. In [35], Yamamoto et al. designed an H_ ∞ _/H_2_ PI observer based on a Linear Parameter-Varying (LPV) framework to observe the system changes. Irmer and Henrichfreise designed a Linear Quadratic Estimation (LQE) to observe the changes in disturbances [36]. However, the analysis of the results in [36] is lacking, making it difficult to evaluate the system’s performance. In [37], Jung and Kim designed an adaptive disturbance observer to estimate changes in crosswinds, which are considered external disturbances. Some other DOs, which are applied to the control mechanism of EPAS systems, have also been mentioned in [38–40]. However, their errors are significant, and the overshooting phenomenon still exists. Several other observers should be referred to [30,31,40,41]. Some applications of intelligent control for EPAS systems have been presented in [42–44]. However, the structure of these algorithms is highly dependent on the designer’s point of view.

Some modern control systems that perform well in automotive mechatronics systems should be referred to in [45,46]. Several applications in EPS system control are presented in [47–50].

### 1.2. Power-assisted characteristic curve overview

The performance of the EPAS system is highly dependent on the characteristics of the ideal assisted curves, which are designed in advance. It is not easy to find the exact variation laws of these curves. In [51], Lee et al. proposed using unsaturated linear curves to generate the ideal reference signal. However, the variation of the assisted torque is considerable. Most previously published studies used saturated linear curves, ensuring the system’s stability under certain conditions. In addition, the mathematical model of these characteristic curves is quite simple, which provides straightforward access [8,17–21,23,34,52]. In [53], Li et al. introduced saturated quadratic curves to reduce increased assisted torque. However, the smoothness of the phase transition remains unresolved. The nonlinear curve, proposed in [25], causes an overshoot in the value as the hand torque increases, which is dangerous for the vehicle during operation. In [54], Guan et al. proposed using different assisted maps for driving modes, including comfortable and sporty styles. The study by Li et al. utilized the ideal assisted multi-map for improving the system adaptation [55]. In addition, the influence of road conditions and adhesion coefficients was also considered when establishing the assisted characteristic curves [56]. However, they are still saturated linear curves facing many limitations [55,56].

### 1.3. Research gaps and key contributions

Research on EPAS control has achieved some results in some instances. However, they still face some drawbacks. First, the performance of traditional control algorithms (such as PID, LPV, and LQR) is not guaranteed for nonlinear systems or systems subject to adverse external influences (external disturbances). Regarding PID control, overshooting and undershooting still occur even when GA, PSO, and ACO techniques have optimized the control parameters. Second, directly measuring multiple state variables causes sensor noise, increasing the steady-state error. As a result, the control quality is degraded. Third, simplifying the mathematical equations of the BSC mechanism causes significant errors compared to the actual results. Fourth, the effect of chattering still exists when the SMC technique is applied to control the system, causing adverse effects on the system. Finally, the design of inappropriate ideal-assisted curves can cause instability under various operating conditions. These are urgent issues that still exist.

This article is motivated by solving the above issues. This work proposes designing a new robust control mechanism that combines SMC and ADRC techniques. The innovation of the control mechanism lies in designing a nonlinear TD and ESO instead of using conventional linear mathematical models. This brings hope in eliminating the influence of nonlinear disturbance and chattering phenomenon, thus improving the system’s performance. In addition, the ideal-assisted curve shapes are innovated to ensure the vehicle’s stability and maintain smoothness and comfort under different conditions, which is considered the second new contribution. To the best of the authors’ knowledge, these innovations have never been presented before.

The structure of the article is organized into four sections. A state-of-the-art review and work motivation are presented in the Introduction section. The innovative mathematical model of the system is introduced in the second section. The third section presents and discusses the simulation results. Finally, the conclusion section provides evaluation perspectives and future work directions.

## 2. Mathematical models

This section presents the mathematical model of the EPAS system and the innovation control mechanism.

### 2.1. EPAS model

**Fig 1:**
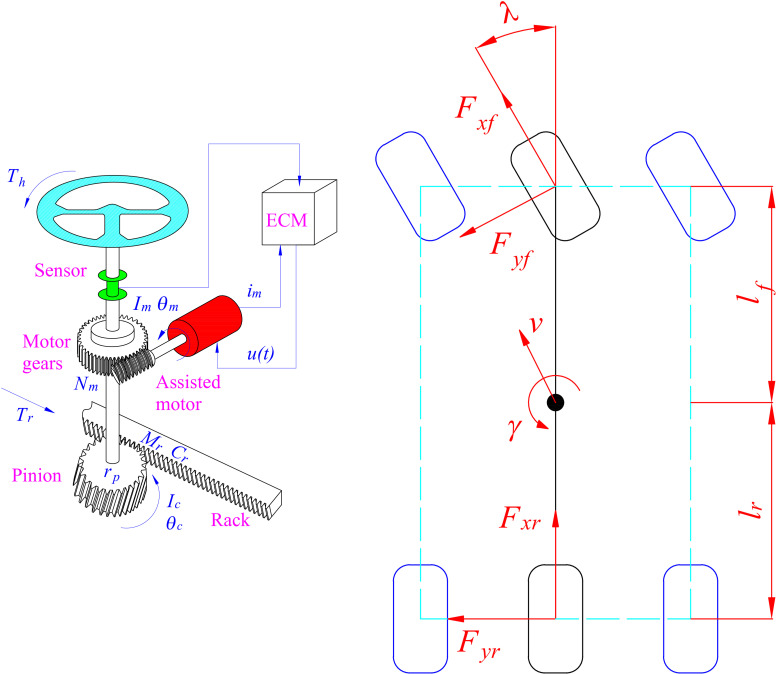
System models. (a) EPAS model. (b) Vehicle model.


The dynamic model of the actuator of the EPAS system is referred to in [25]. Mathematical derivation is summarized as follows (1) and (2). These equations consider the effects of friction and elastic deformation in the steering column. These symbols are mentioned in Fig 1a with specific meanings as follows: *θ*_*c*_ is steering column angle, *θ*_*m*_ is steering motor angle, *I*_*c*_ is column inertia moment, *K*_*c*_ is column stiffness, *C*_*c*_ is column damping, *T*_*h*_ is hand torque, *N*_*m*_ is motor gear ratio, *K*_*t*_ is torque coefficient, *i*_*m*_ is motor current, and *T*_*r*_ is resistant torque.

$$I_{c}{\overset{"}{\theta }}_{c}+C_{c}{\dot{\theta }}_{c}+K_{c}\left(\theta_{c}-\frac{\theta_{m}}{N_{m}}\right)=T_{h}$$ (1)

$$I_{eq}{\overset{"}{\theta }}_{m}+C_{eq}{\dot{\theta }}_{m}+\frac{K_{c}+K_{r}r_{p}^{2}}{N_{m}^{2}}\theta_{m}=\frac{K_{c}}{N_{m}}\theta_{c}+K_{t}i_{m}-\frac{T_{r}}{N_{m}}$$ (2)

*I*_*eq*_ and *C*_*eq*_ are the equivalent moment of inertia and equivalent damping, respectively, and are determined according to (3) and (4), where *M*_*r*_ is rack mass, *r*_*p*_ is pinion radius, and *C*_*r*_ is rack damping.


$$I_{eq}=I_{m}+M_{r}\frac{r_{p}^{2}}{N_{m}^{2}}$$ (3)

$$C_{eq}=C_{m}+C_{r}\frac{r_{p}^{2}}{G_{m}^{2}}$$ (4)

The dynamic equation of a DC motor is expressed as (5), where *R*_*m*_ is motor resistance, *L*_*m*_ is motor inductance, and *u*(*t*) is the control signal.


$$K_{t}{\dot{\theta }}_{m}+L_{m}{\dot{i}}_{m}+R_{m}i_{m}=u\left(t\right)$$ (5)

Resistant torque (*T*_*r*_) consists of two components: endogenous resistance (*T*_*ir*_) and exogenous resistance (*T*_*er*_), which are determined by (6). The endogenous resistance component is generated during steering. It is approximately calculated by equation (7), where *l*_*n*_ is knuckle arm length, *l*_*c*_ is caster trail, *β*_*cas*_ is caster angle, and *β*_*kin*_ is kingpin angle. Exogenous resistance includes road disturbance and other influencing factors, which are considered known inputs in the calculation process. The above method for determining resistance torque is referred to in [25,34].


$$T_{r}=T_{ir}+T_{er}$$ (6)

$$T_{ir}\approx r_{p}l_{c}F_{y1}\frac{cos^{2}\left(\beta_{kin}\right)cos^{2}\left(\beta_{cas}\right)}{l_{n}}$$ (7)

The longitudinal and lateral dynamics of the vehicle are described in (8) and (9), respectively. Equation (10) explains the relationship between the yaw rate and the tire forces. These equations are derived from a linear dynamic model (Fig 1b), which is used to determine the tire forces. The symbols used include *M* is vehicle mass, *v*_*x*_ is longitudinal velocity, *v*_*y*_ is lateral velocity, *γ* is yaw angle, *λ* is steering angle, *F*_*xf*_ is front longitudinal tire force, *F*_*xr*_ is rear longitudinal tire force, *F*_*yf*_ is front lateral tire force, *F*_*yr*_ is rear lateral tire force, *I*_*γ*_ is yaw inertia moment, *l*_*f*_ is front axle distance, and *l*_*r*_ is rear axle distance.


$$M\left({\dot{v}}_{x}-\dot{\gamma }v_{y}\right)=F_{xf}\text{cos}\lambda +F_{xr}-F_{yf}\sin\lambda$$ (8)

$$M\left({\dot{v}}_{y}+\dot{\gamma }v_{x}\right)=F_{yf}\text{cos}\lambda +F_{yr}+F_{xf}\sin\lambda$$ (9)

$$I_{\gamma }\overset{"}{\gamma }=l_{f}\left(F_{xf}\sin\lambda +F_{yr}\text{cos}\lambda \right)-l_{r}F_{yr}$$ (10)

In conclusion, the dynamics of the EPS system are described by equations (1) to (5), while the vehicle dynamics are determined by equations (6) to (10).

### 2.2. Control model

The work aims to design a controller such that the output signals track the reference signals with minimal errors. The ideal EPAS model determines the reference signals controlled by the ideal assisted curves. We propose designing the ideal curves using a complex nonlinear function, as in equation (11), where *k*_*a*_ and *k*_*g*_ are the curve coefficients and *e* is Euler’s number. A quadratic function *k*(*v*), which depends on the vehicle velocity, is presented in (12), with *k*_*vi*_ being empirical coefficients.


$$T_{a}\left(v,T_{h}\right)=k_{v}\left(v\right)k_{g}\left(\frac{1}{1+e^{-k_{a}T_{h}^{3}}}-\frac{1}{1+e^{k_{a}T_{h}^{3}}}\right)$$ (11)

$$k_{v}\left(v\right)=k_{v1}v^{2}+k_{v2}v+k_{v3}$$ (12)

**Fig 2:**
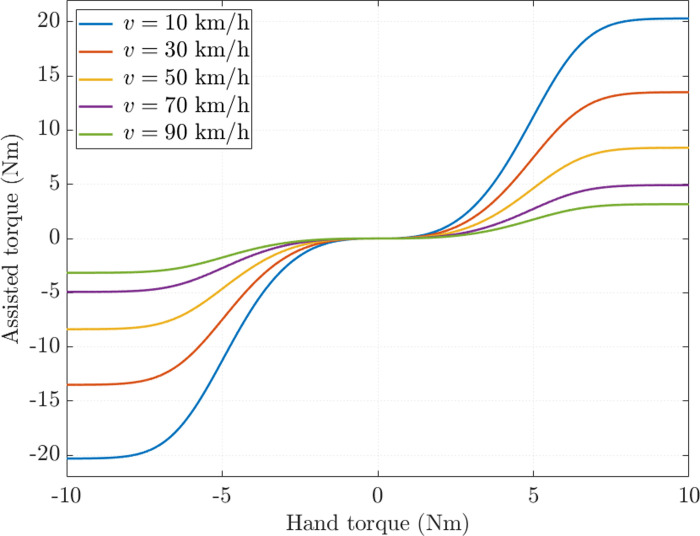
Ideal assisted torque.

The characteristic curves designed according to (11) ensure smooth phase transitions, avoid overshoot when hand torque increases abruptly, and limit the effects of premature convergence (Fig 2). When the hand torque is small, the increased assisted torque is negligible. When the speed decreases, the value of assisted torque increases nonlinearly according to the quadratic function mentioned in (12). This is a unique type of nonlinear curve used for the first time to generate ideal values for the EPAS system.

The robust control algorithm is designed in this work to control the automotive EPAS system. This control mechanism is formed based on SMC and nonlinear ADRC techniques. The state variables of the mathematical model are arranged in the order as shown in (13), including steering column angle (*x*_1_), steering column angular velocity (*x*_2_), steering motor angle (*x*_3_), steering motor angular velocity (*x*_4_), and motor current (*x*_5_).


$$\left[\begin{array}{ccccc} x_{1} & x_{2} & x_{3} & x_{4} & x_{5} \end{array}\right]=\left[\begin{array}{ccccc} \theta_{c} & {\dot{\theta }}_{c} & \theta_{m} & {\dot{\theta }}_{m} & i_{m} \end{array}\right]$$ (13)

We get (14) to (18) by taking the state variables’ derivatives.


$${\dot{x}}_{1}=x_{2}$$ (14)

$${\dot{x}}_{2}=-\frac{K_{c}}{I_{c}}x_{1}-\frac{C_{c}}{I_{c}}x_{2}+\frac{K_{c}}{I_{c}N_{m}}x_{3}+\frac{T_{h}}{I_{c}}$$ (15)

$${\dot{x}}_{3}=x_{4}$$ (16)

$${\dot{x}}_{4}=\frac{K_{c}}{I_{eq}N_{m}}x_{1}-\frac{K_{c}+K_{r}r_{p}^{2}}{I_{eq}N_{m}^{2}}x_{3}-\frac{C_{eq}}{I_{eq}}x_{4}+\frac{K_{t}}{I_{eq}}x_{5}-\frac{T_{r}}{I_{eq}N_{m}}$$ (17)

$${\dot{x}}_{5}=-\frac{K_{t}}{L_{m}}x_{4}-\frac{R_{m}}{L_{m}}x_{5}+\frac{1}{L_{m}}u\left(t\right)$$ (18)

In this work, we propose the design of the NTD to smooth the reference signal and eliminate the influence of disturbances. In addition, the state variables and system disturbances are estimated by the NESO to reduce the influence of sensor noise and the system cost. The design of nonlinear mathematical models is highly effective in controlling the system and eliminating the influence of external disturbances.

It is assumed that only the sensor measures the first state variable (*θ*_*c*_). The error between the directly measured and observed signals is denoted by *e*_*β*_, according to (19). The mathematical model of NESO is described by equations (20) to (25), where the resistant torque is determined through an augmented variable (26).


$$e_{\beta }=x_{1}-{\hat{x}}_{1}$$ (19)

$${\dot{\hat{x}}}_{1}={\hat{x}}_{2}+\beta_{1}$$ (20)

$${\dot{\hat{x}}}_{2}=-\frac{K_{c}}{I_{c}}{\hat{x}}_{1}-\frac{C_{c}}{I_{c}}{\hat{x}}_{2}+\frac{K_{c}}{I_{c}N_{m}}{\hat{x}}_{3}+\frac{T_{h}}{I_{c}}+\beta_{2}$$ (21)

$${\dot{\hat{x}}}_{3}={\hat{x}}_{4}+\beta_{3}$$ (22)

$${\dot{\hat{x}}}_{4}=\frac{K_{c}}{I_{eq}N_{m}}{\hat{x}}_{1}-\frac{K_{c}+K_{r}r_{p}^{2}}{I_{eq}N_{m}^{2}}{\hat{x}}_{3}-\frac{C_{eq}}{I_{eq}}{\hat{x}}_{4}+\frac{K_{t}}{I_{eq}}{\hat{x}}_{5}-\frac{1}{I_{eq}N_{m}}{\hat{x}}_{6}+\beta_{4}$$ (23)

$${\dot{\hat{x}}}_{5}=-\frac{K_{t}}{L_{m}}{\hat{x}}_{4}-\frac{R_{m}}{L_{m}}{\hat{x}}_{5}+\frac{1}{L_{m}}u\left(t\right)+\beta_{5}$$ (24)

$${\dot{\hat{x}}}_{6}=\beta_{6}$$ (25)

$${\hat{x}}_{6}=T_{r}$$ (26)

The observed gains (*β*_*i*_) are calculated using a nonlinear model, presented in (27), where *l*_*i*_ are observed coefficients. The nonlinear function, fal(.) function, is described in (28), where *α*_*i*_ are tuning parameters, and *δ*_*i*_ are threshold parameters.


$$\beta_{i}=l_{i}\text{fal}\left(e_{\beta };\alpha_{i};\delta_{i}\right)$$ (27)

$$\text{fal}\left(e_{\beta };\alpha_{i};\delta_{i}\right)= \{\begin{array}{cc} \frac{e_{\beta }}{\delta_{i}^{1-\alpha_{i}}} & \left|e_{\beta }\right|\leq \delta_{i}\\ \text{sign}\left(e_{\beta }\right){\left|e_{\beta }\right|}^{\alpha_{i}} & \left|e_{\beta }\right|>\delta_{i} \end{array}$$ (28)

The structure of the NTD is presented in (29) and (30), where *z*_*i*_ are new outputs and *k*_*i*_ are positive constants.


$${\dot{z}}_{1}=z_{2}$$ (29)

$${\dot{z}}_{2}=-k_{z1}e_{z1}-k_{z2}\text{sat}\left(e_{z2}\right)+{\overset{"}{x}}_{3\_ref}=z_{3}$$ (30)

Let *e*_*z*1_ and *e*_*z*2_ be the errors of the NTD, which are explained in detail in (31) and (32), respectively. Equation (33) is obtained by combining (30) with the derivative of (32).


$$e_{z1}=z_{1}-x_{3\_ref}$$ (31)

$$e_{z2}={\dot{e}}_{z1}=z_{2}-{\dot{x}}_{3\_ref}$$ (32)

$${\dot{e}}_{z2}=-k_{z1}e_{z1}-k_{z2}sat\left(e_{z2}\right)$$ (33)

The error between the smoothed and observed signals is denoted by *e*_3_, according to (34). Equations (35) and (36) are obtained by taking the first and second derivatives of *e*_3_, respectively. The *b*_*i*_ symbols used in equation (36) are explained according to (37) to (42).


$$e_{3}=z_{1}-{\hat{x}}_{3}$$ (34)

$${\dot{e}}_{3}={\dot{z}}_{1}-{\dot{\hat{x}}}_{3}=z_{2}-{\hat{x}}_{4}-\beta_{3}$$ (35)

$${\overset{"}{e}}_{3}={\dot{z}}_{2}-{\dot{\hat{x}}}_{4}-{\dot{\beta }}_{3}=z_{3}-\sum_{i=1}^{6}b_{i}{\hat{x}}_{i}-\beta_{4}-{\dot{\beta }}_{3}$$ (36)

$$b_{1}=\frac{K_{c}}{I_{eq}N_{m}}$$ (37)

$$b_{2}=0$$ (38)

$$b_{3}=-\frac{K_{c}+K_{r}r_{p}^{2}}{I_{eq}N_{m}^{2}}$$ (39)

$$b_{4}=-\frac{C_{eq}}{I_{eq}}$$ (40)

$$b_{5}=\frac{K_{t}}{I_{eq}}$$ (41)

$$b_{6}=-\frac{1}{I_{eq}N_{m}}$$ (42)

The controlled object is denoted according to (43). Taking the derivative of *y*, we get (44) to (46). The symbols (*a*_*i*_) mentioned in (46) are explicitly explained according to (47) to (52).


$$y={\hat{x}}_{3}$$ (43)

$$\dot{y}={\hat{x}}_{4}+\beta_{3}$$ (44)

$$\overset{"}{y}=\sum_{i=1}^{6}b_{i}{\hat{x}}_{i}+\beta_{4}+{\dot{\beta }}_{3}$$ (45)

$$\dddot{y}=\sum_{i=1}^{6}a_{i}{\hat{x}}_{i}+\sum_{i=1}^{6}b_{i}\beta_{i}+{\dot{\beta }}_{4}+{\overset{"}{\beta }}_{3}+\frac{K_{t}}{I_{eq}L_{m}}u\left(t\right)=\hat{d}+bu\left(t\right)$$ (46)

$$a_{1}=-\frac{C_{eq}K_{c}}{I_{eq}^{2}N_{m}}$$ (47)

$$a_{2}=\frac{K_{c}}{I_{eq}N_{m}}$$ (48)

$$a_{3}=\frac{C_{eq}\left(K_{c}+K_{r}r_{p}^{2}\right)}{I_{eq}^{2}N_{m}^{2}}$$ (49)

$$a_{4}=-\frac{K_{c}+K_{r}r_{p}^{2}}{I_{eq}N_{m}^{2}}+\frac{C_{eq}^{2}}{I_{eq}^{2}}-\frac{K_{t}^{2}}{I_{eq}L_{m}}$$ (50)

$$a_{5}=-\frac{C_{eq}K_{t}}{I_{eq}^{2}}-\frac{K_{t}R_{m}}{I_{eq}L_{m}}$$ (51)

$$a_{6}=\frac{C_{eq}}{I_{eq}^{2}N_{m}}$$ (52)

Taking the third derivative of (34), we get (53).


$${\dddot{e}}_{3}={\dddot{z}}_{1}-{\dddot{\hat{x}}}_{3}=\dddot{z}-\dddot{y}$$ (53)

**Fig 3:**
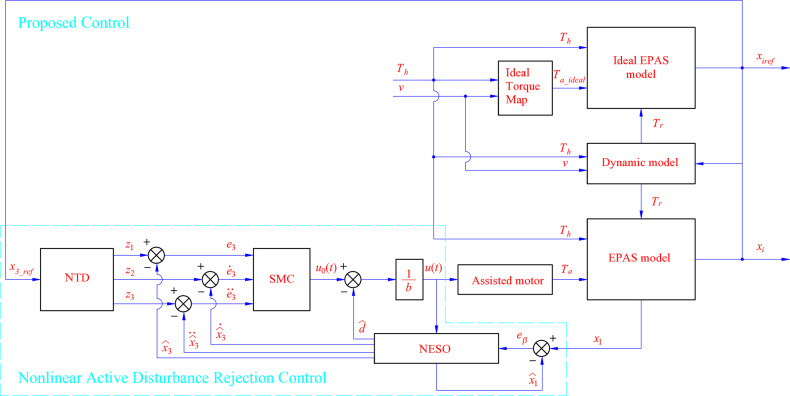
Proposed control scheme.


The state feedback control law is selected according to (54), where *d* is the total disturbance and *u*_0_(*t*) is the initial control signal (Fig 3).

$$u\left(t\right)=\frac{u_{0}\left(t\right)-\hat{d}}{b}$$ (54)

A linear sliding surface (Ω) is chosen according to (55).


$$\Omega ={\overset{"}{e}}_{3}+k_{s1}{\dot{e}}_{3}+k_{s2}e_{3}$$ (55)

Taking the derivative of (55), we get


$$\dot{\Omega }={\dddot{z}}_{1}-\dddot{y}+k_{s1}{\overset{"}{e}}_{3}+k_{s2}{\dot{e}}_{3}$$ (56)

The value of *u*_0_(*t*) is chosen according to (57) to ensure the system’s stability, where *k*_*si*_ are the coefficients.


$$u_{0}\left(t\right)={\dddot{z}}_{1}+k_{s1}{\overset{"}{e}}_{3}+k_{s2}{\dot{e}}_{3}+k_{s3}\text{sat}\left(\Omega \right)$$ (57)

In this article, the theoretical stability of the system is evaluated using Lyapunov stability. The candidate Lyapunov function is presented in (58). This candidate function consists of two components: the tracking error (*e*_*z*_) representing the NTD and the sliding surface (Ω) representing the SMC mechanism. The coefficients *k*_*z*1_ and *k*_*z*2_ are chosen to be positive. As a result, *V*(*x*) is positive-definite for all nonzero *x*.


$$\begin{array}{cc} V\left(x\right)=\frac{1}{2}k_{z1}e_{z1}^{2}+\frac{1}{2}e_{z2}^{2}+\frac{1}{2}\Omega^{2}>0 & \forall x\neq 0 \end{array}$$ (58)

Taking the derivative of *V*(*x*), then combining it with (33), (54), (56), and (57), we get (59). Once *k*_*s*3_ is a positive coefficient, (59) will be negative-definite. Combining (58) and (59), the system is considered theoretically stable.


$$\begin{array}{l} \dot{V}\left(x\right)=k_{z1}e_{z1}{\dot{e}}_{z1}+e_{z2}{\dot{e}}_{z2}+\Omega \dot{\Omega }\\ =k_{z1}e_{z1}e_{z2}+e_{z2}\left[-k_{z1}e_{z1}-k_{z2}\text{sat}\left(e_{z2}\right)\right]+\Omega \left({\dddot{z}}_{1}-\dddot{y}+k_{s1}{\overset{"}{e}}_{3}+k_{s2}{\dot{e}}_{3}\right)\\ =-k_{z2}e_{z2}\text{sat}\left(e_{z2}\right)+\Omega \left\{{\dddot{z}}_{1}-\hat{d}-b\frac{\left[{\dddot{z}}_{1}+k_{s1}{\overset{"}{e}}_{3}+k_{s2}{\dot{e}}_{3}+k_{s3}\text{sat}\left(\Omega \right)\right]-\hat{d}}{b}+k_{s1}{\overset{"}{e}}_{3}+k_{s2}{\dot{e}}_{3}\right\}\\ =\begin{array}{cc} -k_{z2}e_{z2}\text{sat}\left(e_{z2}\right)-k_{s3}\Omega \text{sat}\left(\Omega \right)<0 & \forall x\neq 0 \end{array} \end{array}$$ (59)

Regarding the NESO, the observed coefficients (*l*_*i*_) are chosen by the pole placement method to ensure that the poles of the closed-loop system are always on the left side of the complex plane to ensure the system’s stability.

## 3. Simulation and discussion

**Fig 4:**
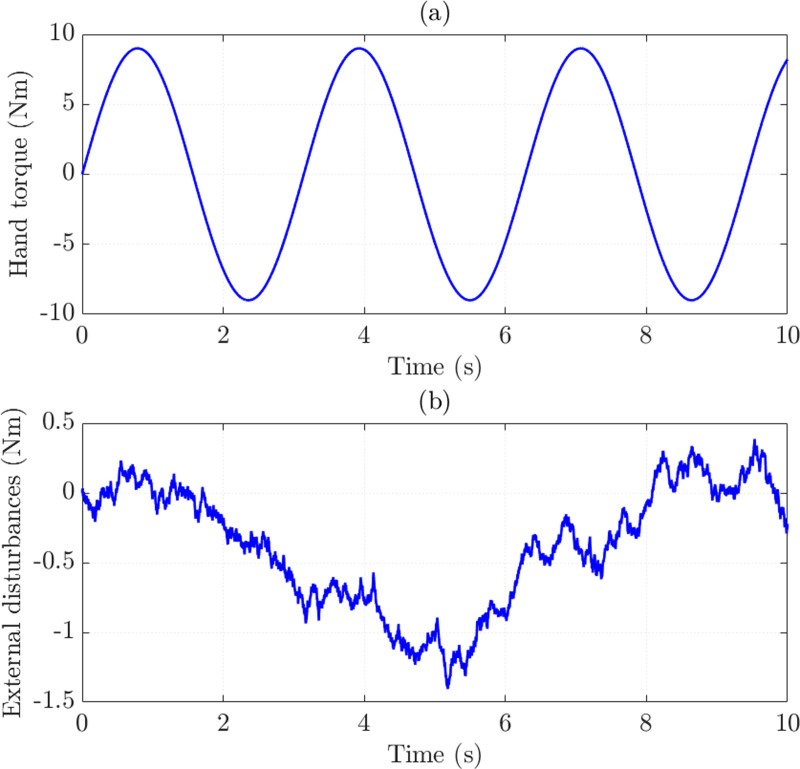
Simulation conditions. (a) Hand torque. (b) External disturbances.

Numerical simulations are performed to verify the performance of the proposed control mechanism. Hand torque (*T*_*h*_) is considered a known input, as shown in Fig 4a. The effect of road disturbances is presented in Fig 4b.

The investigation is carried out in two different cases. In the first case, the vehicle steers at low speed (*v*_1_ =  30 km/h). The second case uses a higher speed value (*v*_2_ =  70 km/h). The changes in steering column angle (SCA), steering column angular velocity (SCAV), steering motor angle (SMA), and steering motor angular velocity (SMAV) are considered outputs. The assisted performance is evaluated through the steering motor current and assisted torque. The results obtained by the proposed controller are compared with Linear Quadratic Tracking (LQT), PI, and SMC controllers to verify the quality and superiority of the robust control method, which is introduced in this work.

### 3.1. Simulation results

#### The first case.

**Fig 5:**
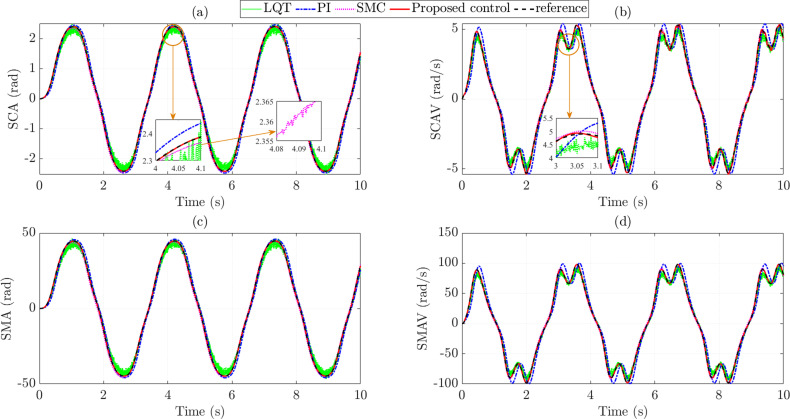
State variables (*v*_1_ =  30 km/h). (a) Steering column angle. (b) Steering column angular velocity. (c) Steering motor angle. (d) Steering motor angular velocity.

***v*_1_ = 30 km/h,** The simulation results in the first case are shown by the subplots in Fig 5. Fig 5a provides information on the changes in SCA over time. Generally, the results obtained from the four controllers tend to change according to the steering law proposed in Fig 4a. The steady-state error of the PI controller is 0.139 rad, which is 1.18 times higher than that of the LQT controller. The window subplot shows that the LQT controller’s output signal is negatively affected by sensor noise, causing a deterioration in control performance. In this case, the RMS error of the SMC controller is 0.057 rad (corresponding to 3.452%). In addition, the output signal of the SMC controller is heavily affected by chattering. Once the proposed technique replaces the above control techniques, the steady-state error is greatly reduced to 0.002 rad (0.127%). The effects of chattering and sensor noise phenomena are eliminated once this technique is applied to control the EPAS system. As shown in Fig 5b, the SCAV signal obtained from the PI controller is overshooting, causing the RMS error to increase to 17.166%. Under the influence of sensor noise and chattering, the RMS errors of the LQT and SMC controllers still maintain relatively large values, 8.635% and 2.727%, respectively. The tracking ability of the SCAV signal is greatly improved once the EPAS system is controlled by the proposed controller, which is formed based on the combination of SMC and nonlinear ADRC techniques, causing the RMS error to decrease to 1.963%.

Although SMA’s value is more significant than SCA’s, their changing trends are similar. According to the simulation results shown in Fig 5c, the steady-state error of the proposed controller is only 0.099%, while those of the LQT, PI, and SMC controllers are 7.385%, 8.577%, and 3.536%, respectively. The variation in SMAV value is illustrated in Fig 5d with five different types of curves. Overall, the signal obtained from the proposed controller closely follows the reference signal with a negligible error (0.370%). Under the influence of overshoot, the RMS error of the PI controller increases to 10.981rad/s, which is 17.029%. Although the LQT technique can control MIMO systems, the influence of sensor noise still exists, causing the steady-state error to increase to 8.706%. The deviation between the SMC and reference signals is 2.696%, caused by the chattering phenomenon.

**Fig 6:**
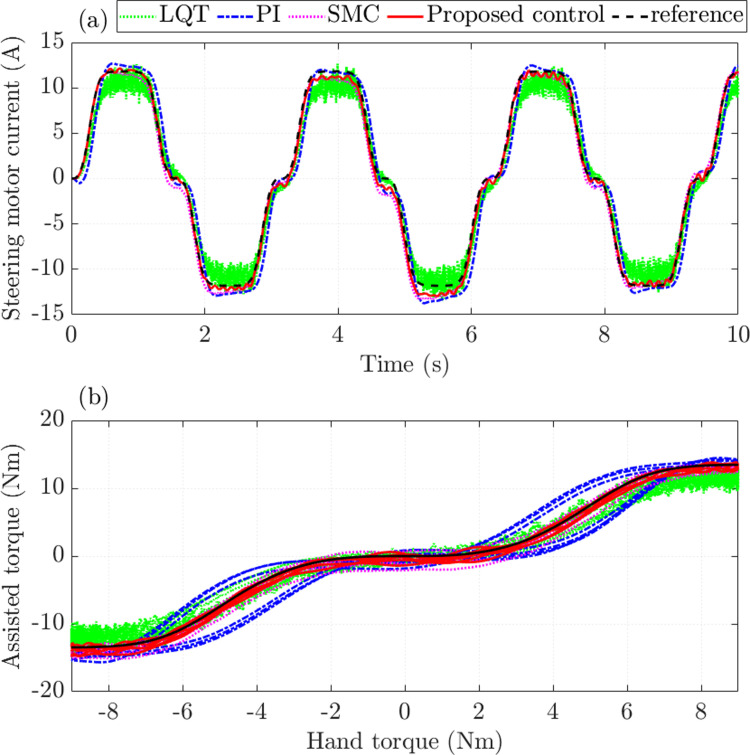
Assisted performance (*v*_1_ =  30 km/h). (a) Steering motor current. (b) Assisted torque.

The quality of the controllers is evaluated through the steering performance. Fig 6a shows that the average power consumption of the PI controller is the largest, with an RMS error of up to 19.394% compared to the ideal controller. The error of the LQT controller is slightly smaller than the PI controller’s, which is 14.934%. Under the effective control of the SMC mechanism, the steady-state error of the steering motor current is only 9.266%. Once the SMC technique is combined with the nonlinear ADRC technique to become the proposed controller, the energy consumption efficiency is significantly improved, causing the error to decrease to 6.061%. In addition, overshoot, sensor noise, and chattering are almost eliminated once the proposed controller is utilized to control the steering system.

Fig 6b shows the relationship between assisted torque and hand torque. In general, as the hand torque value increases, the assisted torque value also increases. This change is nonlinear and consistent with the rule proposed in Fig 2. The simulation results show that LQT and PI controllers’ errors are significant, while SMC and proposed control errors are much more minor.

**Fig 7:**
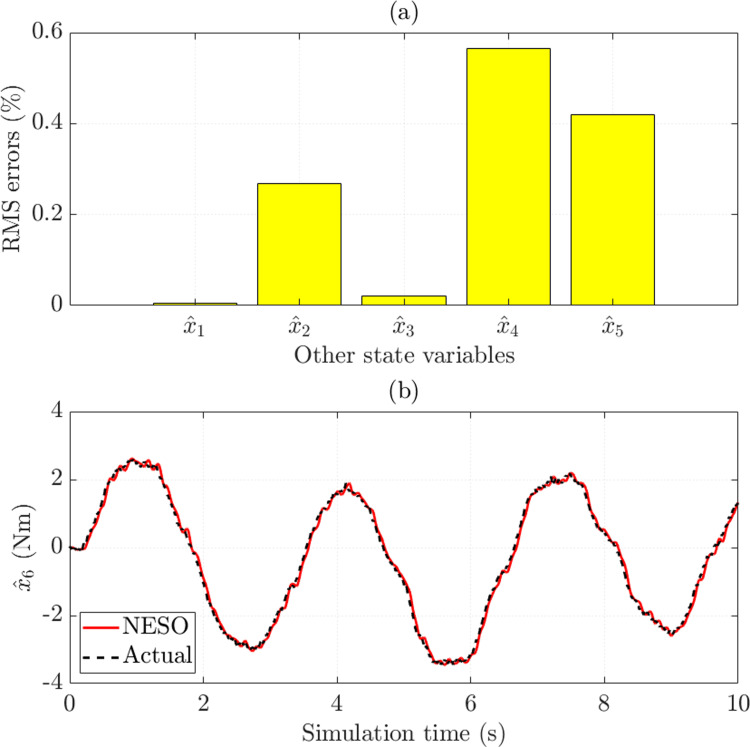
Observed errors (*v*_1_ =  30 km/h). (a) RMS errors. (b) Augmented variable error.

In this work, the NESO estimates the state variables instead of directly measured by expensive sensors. This contributes to reducing the operating cost and eliminating the influence of sensor noise. The simulation results reveal that the observed errors of SCA (*x*_1_) and SMA (*x*_3_) are minimal, 0.006% and 0.022%, respectively. In addition, the RMS errors of the remaining state variables do not exceed 0.6% (Fig 7a). The variation of the system disturbance over time is estimated through the augmented variable. With an RMS error of only 6.922% and a mean error of only 1.485%, the prediction results in Fig 7b show that the observed signal through NESO is exceptionally close to the actual signal.

**Fig 8:**
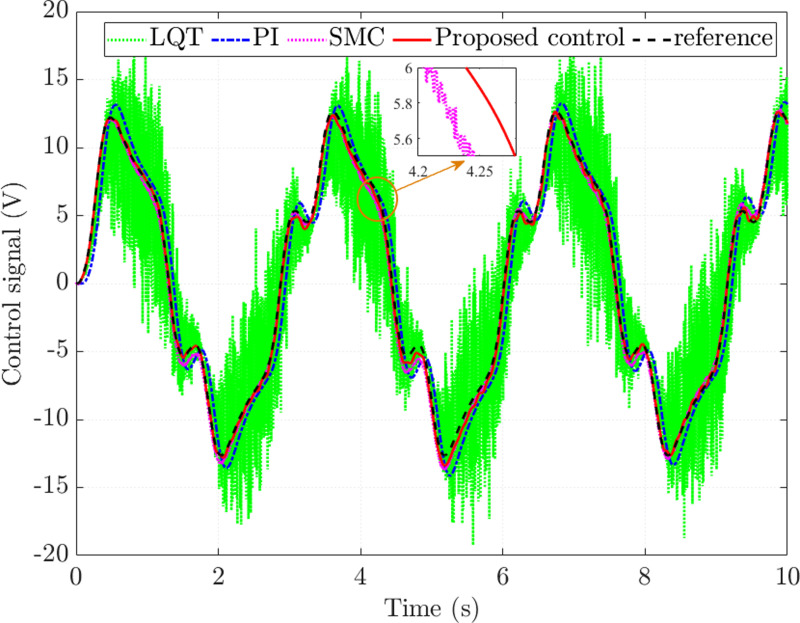
Control signal (*v*_1_ =  **30 km/h).**

The control signal generated by the proposed controller closely follows the reference signal with an RMS error not exceeding 4.014%, while the steady-state error of the sliding mode controller is 6.511% (Fig 8). Compared with the reference value, the errors of the conventional controllers are significant, being 30.684% (LQT) and 19.583% (PI), respectively. These results demonstrate that the proposed controller effectively observes the state variables with minor errors.

Table 1 below provides complete information on the simulation results for the first case. One thing to note is that these data have been rounded.

**Table 1:** RMS percentage errors (*v*_1_ =  30 km/h).

	PI	LQT	SMC	Proposed control	Observed
SCA	8.418	7.146	3.452	0.127	0.006
SCAV	17.166	8.635	2.727	1.963	0.268
SMA	8.577	7.385	3.536	0.099	0.022
SMAV	17.029	8.706	2.696	0.370	0.565
Motor current	19.394	14.934	9.266	6.061	0.420
Control signal	19.583	30.684	6.511	4.014	

#### The second case: *v*_2_ = 70 km/h.

Fig 2 shows that the assisted performance changes as the vehicle speed changes. Therefore, it is necessary to investigate the dynamic behavior of the EPAS system at higher speed. The speed *v*_2_ =  70 km/h is the investigated value in this case.

**Fig 9:**
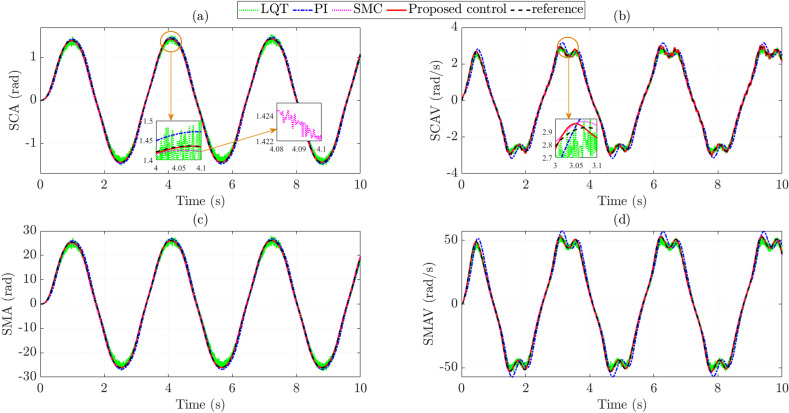
State variables (*v*_2_ =  70 km/h). (a) Steering column angle. (b) Steering column angular velocity. (c) Steering motor angle. (d) Steering motor angular velocity.

The SCA, SCAV, SMA, and SMAV changes when the vehicle moves at *v*_2_ =  70 km/h are depicted in the subplots in Fig 9. Generally, the values obtained in this case are smaller than those in the first case (*v*_1_ =  30 km/h). According to the article’s findings, the steady-state error of SCA is the largest when the EPAS system is controlled by the conventional PI controller (5.911%) (Fig 9a). Compared with PI control, LQT control provides better control efficiency for MIMO systems, reducing RMS error to 5.452%, slightly higher than that of SMC (2.975%). Fig 9a shows that the SCA signal obtained from the LQT controller is strongly affected by sensor noise. In contrast, the signal obtained from the SMC controller has to undergo chattering, leading to system performance degradation. In contrast, these problems are almost eliminated once the above control techniques are replaced with the technique proposed in this article. As a result, the steady-state error is reduced to 0.002 rad, corresponding to 0.247%.

The difference in SCA errors is insignificant, while the difference in SCAV errors is considerable. According to the simulation results shown in Fig 9b, the PI error is up to 11.344%, caused by an overshoot. The RMS error of LQT control is only about 54.94% of that of conventional PI. Compared with the two classical control techniques mentioned above, the proposed technique provides superior performance in system control. This is confirmed by the tracking error (3.404%). The remaining results (SMA and SMAV) in Figs 9c and 9d confirm that the tracking error of the proposed controller is the smallest compared to the other controllers. In addition, the effects of negative phenomena (such as overshoot, chattering, and sensor noise) are almost eliminated once this control technique is utilized.

**Fig 10:**
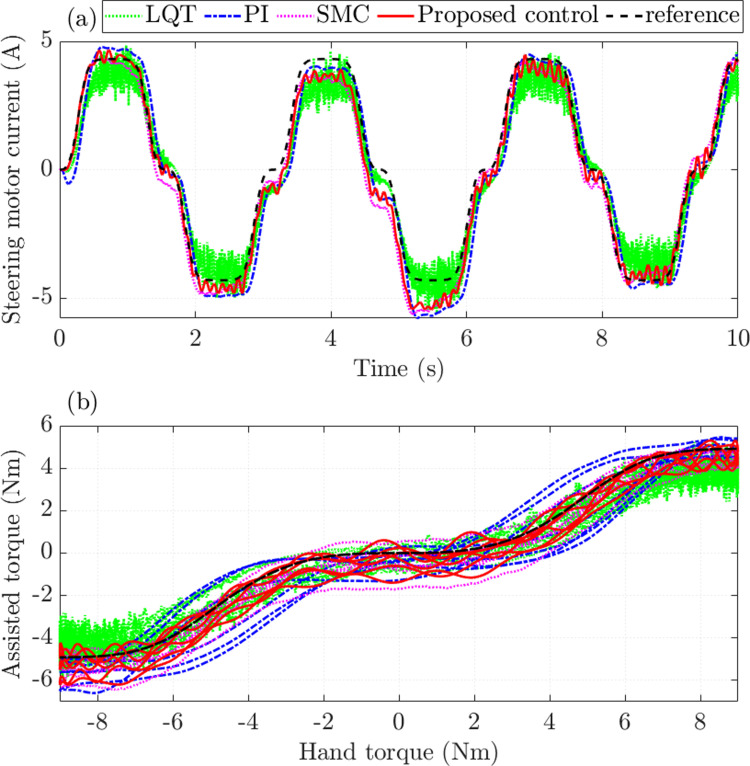
Assisted performance (*v*_2_ =  70 km/h). (a) Steering motor current. (b) Assisted torque.

The assisted steering performance of the EPAS system deteriorates as the speed increases, which is in line with the rule proposed in Fig 2. Therefore, the steering motor current errors also increase. Under the influence of sensor noise, the RMS error of the LQT control is up to 21.930%, causing significant energy loss (Fig 10a). The fundamental reason for this phenomenon is that too many physical sensors are used to measure the output state variables. The steady-state error of the classical PI controller is slightly higher than that of the LQT controller. Generally, the current signals obtained by these two controllers do not closely follow the reference signal. In this case, the error obtained by the proposed control is significantly lower (16.501%), while the error obtained by the SMC is 19.323%. Although system performance is ensured by leveraging the SMC technique, the effects of chattering persist at times.

The characteristic curves obtained from SMC and proposed control follow the target signal with minor errors, while LQT and PI control errors are more significant (Fig 10b).

**Fig 11:**
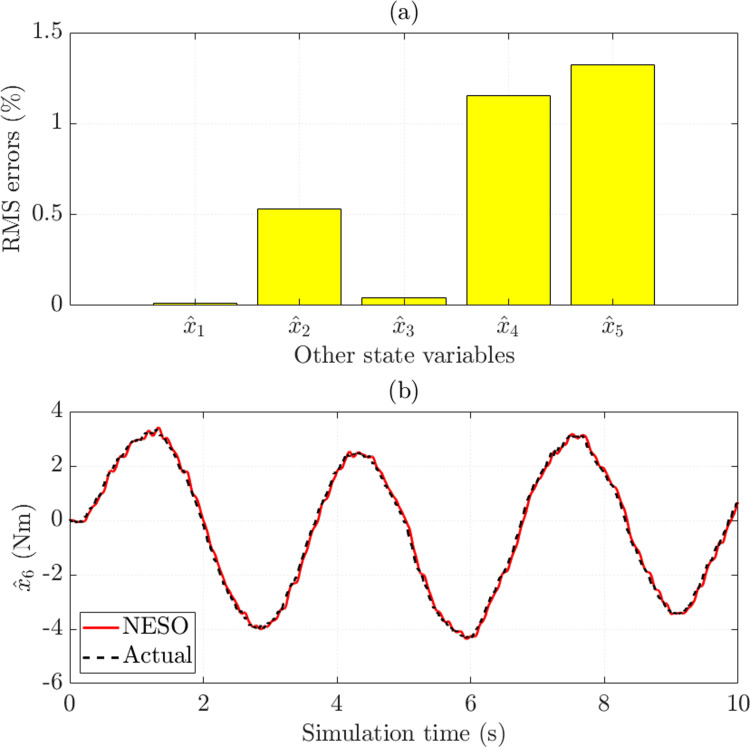
Observed errors (*v*_2_ =  70 km/h). (a) RMS errors. (b) Augmented variable error.

The observed errors of the state variables are presented in Fig 11a. In general, these values are pretty small, not exceeding 1.4%. In the second case, the observed disturbance follows the target signal with high accuracy, up to about 94% (Fig 11b).

**Fig 12:**
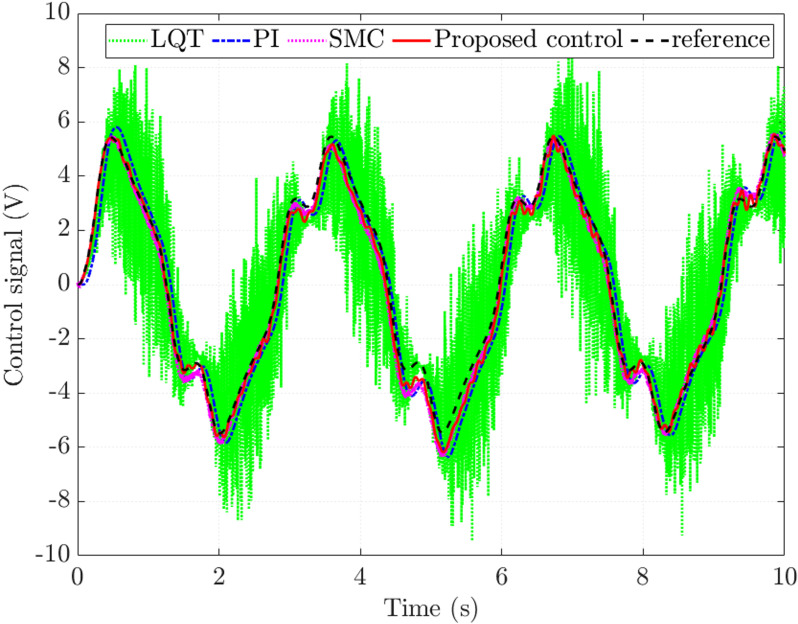
Control signal (*v*_2_ **=**  **70 km/h).**

The simulation results presented in Fig 12 show that the control signal generated by the proposed controller is closer to the reference signal than the other controllers. The steady-state error in this case is more significant than in the first case (*v*_1_ =  30 km/h) because of the system performance degradation as the speed increases. Table 2 lists the rounded simulation results for the second case.

**Table 2:** RMS percentage errors (*v*_2_ =  70 km/h).

	PI	LQT	SMC	Proposed control	Observed
SCA	5.911	5.452	2.975	0.247	0.011
SCAV	11.344	6.232	2.707	3.404	0.529
SMA	6.151	5.759	3.110	0.211	0.044
SMAV	11.490	6.406	2.733	0.639	1.155
Motor current	25.578	21.930	19.323	16.501	1.325
Control signal	19.286	38.159	11.146	9.257	

### 3.2. Discussion

The simulation results in two cases show that the proposed algorithm performs better than other control algorithms for controlling the vehicle’s steering system. This is verified through the steady-state errors of the output variables. In general, the RMS error of the proposed controller is tiny, while the figures for the remaining controllers are significant. Furthermore, the problems of overshoot (PI), chattering (SMC), and sensor noise (LQT) are mostly fixed when the proposed controller is used instead of the previous ones. Compared with some other studies [8,19,24,39,57], the steady-state errors of the state variables are significantly improved.

The energy consumption of the proposed controller is less than that of the traditional controllers. This is validated through the simulation results mentioned above. Compared with other control algorithms [58,59], the energy-saving effect of the proposed control mechanism has been significantly improved. In addition, the NESO provides high accuracy in observing the state variables and system disturbances. The observed error of the NESO, which is designed in this work, is much smaller than that of the conventional observer [30,31,39,40,60].

The ideal characteristic curves proposed in this work provide smooth and comfortable operation. The innovation in designing the ideal characteristic curves can solve several existing problems related to the loss of smoothness during phase transition [8,17–21,23,34,52,55,56,60], unsaturation [45], and sudden increase in value during steering [25].

### 3.3. Future work

Experimental tests need to be conducted shortly to validate the effectiveness of the proposed algorithm. While the current study primarily relies on simulation results, preliminary validation through Hardware-in-the-Loop (HIL) testing is essential to determine the efficiency of the proposed control scheme. The proposed HIL testing procedure is as follows:

**Preparation:** Set up a test bench with a vehicle model equipped with an EPS system.

**Sensor Installation:** Equip the system with necessary sensors, including a steering torque sensor, steering angle sensor, vehicle speed sensor, and others to collect real-time data.

**Algorithm Deployment:** Implement the proposed control algorithm on a real-time device (MicroAutobox II).

**Experimental Execution:** Conduct experiments under predefined conditions, ensuring that the generated hand torque and external disturbances match those used in simulations.

**Performance Evaluation:** Measure essential parameters and compare the results with reference values to assess the system’s effectiveness.

These preliminary experiments will offer valuable insights into the real-time performance of the proposed controller, allowing for refinements and optimizations before large-scale deployment. Future research will continue with extended HIL testing and real-world driving evaluations to determine the effectiveness of the control strategy.

## 4. Conclusion

This article presents the design of a robust nonlinear algorithm, which is formed based on the combination of SMC and nonlinear ADRC techniques. The work aims to reduce the steady-state error and enhance the tracking performance while eliminating the adverse effects on the system. In addition, new characteristic curves are proposed in this work to improve driving comfort and reduce vehicle instability under different conditions. Simulation results show that the proposed algorithm performs better in controlling the EPAS system than the existing algorithms. This is verified by the significant reduction of the RMS errors of the state variables and the elimination of the influence of overshoot, sensor noise, and chattering. Finally, the state variables estimated by the NESO have high accuracy, improving the system’s control efficiency.

The work done in this article still faces some remaining challenges. First, the steady-state error must be gradually reduced to zero, especially the steering motor current error. Second, the augmented variable, utilized to observe the disturbance, must be calculated more accurately to improve the system’s performance. Third, the sliding surface of the SMC mechanism should be dynamically tuned to reduce the influence of chattering and improve the tracking efficiency. The first two issues can be addressed by applying an optimization technique to find the ideal values for the controller. Designing a fuzzy rule to tune the sliding surface is an effective solution for the third issue. Finally, practical experiments or HIL tests need to be conducted to verify the effectiveness of the proposed algorithm. These drawbacks will be addressed in future works.
